# The Regulations of Deubiquitinase USP15 and Its Pathophysiological Mechanisms in Diseases

**DOI:** 10.3390/ijms18030483

**Published:** 2017-02-24

**Authors:** Chon-Kit Chou, Yu-Ting Chang, Michal Korinek, Yei-Tsung Chen, Ya-Ting Yang, Steve Leu, I-Ling Lin, Chin-Ju Tang, Chien-Chih Chiu

**Affiliations:** 1 Graduate Institute of Natural Products, Kaohsiung Medical University, Kaohsiung 807, Taiwan; fatchou1988@hotmail.com (C.-K.C.); mickorinek@hotmail.com (M.K.); 2 Department of Biotechnology, Kaohsiung Medical University, Kaohsiung 807, Taiwan; c.jasmine8306@gmail.com (Y.-T.C.); tinayang101@gmail.com (Y.-T.Y.); st.leu.tw@gmail.com (S.L.); candace0602@gmail.com (C.-J.T.); 3 Department of Medicine, Yong Loo Lin School of Medicine, National University of Singapore, Singapore 117, Singapore; mdccyt@nus.edu.sg; 4 Institute for Translational Research in Biomedicine, Kaohsiung Chang Gung Memorial Hospital, Kaohsiung 807, Taiwan; 5 Department of Medical Laboratory Science and Biotechnology, Kaohsiung Medical University, Kaohsiung 807, Taiwan; linili@cc.kmu.edu.tw; 6 Center of Excellence for Environmental Medicine, Kaohsiung Medical University, Kaohsiung 807, Taiwan; 7 Graduate Institute of Medicine, College of Medicine, Kaohsiung Medical University, Kaohsiung 807, Taiwan; 8 Translational Research Center, Cancer Center and Department of Medical Research, Kaohsiung Medical University Hospital, Kaohsiung 807, Taiwan; 9 Department of Biological Sciences, National Sun Yat-sen University, Kaohsiung 804, Taiwan

**Keywords:** deubiquitinase, ubiquitin-specific protease 15, TGF-β, IκBα, cancer-related signaling networks, Parkinson’s disease

## Abstract

Deubiquitinases (DUBs) play a critical role in ubiquitin-directed signaling by catalytically removing the ubiquitin from substrate proteins. Ubiquitin-specific protease 15 (USP15), a member of the largest subfamily of cysteine protease DUBs, contains two conservative cysteine (Cys) and histidine (His) boxes. USP15 harbors two zinc-binding motifs that are essential for recognition of poly-ubiquitin chains. USP15 is grouped into the same category with USP4 and USP11 due to high degree of homology in an N-terminal region consisting of domains present in ubiquitin-specific proteases (DUSP) domain and ubiquitin-like (UBL) domain. USP15 cooperates with COP9 signalosome complex (CSN) to maintain the stability of cullin-ring ligase (CRL) adaptor proteins by removing the conjugated ubiquitin chains from RBX1 subunit of CRL. USP15 is also implicated in the stabilization of the human papillomavirus type 16 E6 oncoprotein, adenomatous polyposis coli, and IκBα. Recently, reports have suggested that USP15 acts as a key regulator of TGF-β receptor-signaling pathways by deubiquitinating the TGF-β receptor itself and its downstream transducers receptor-regulated SMADs (R-SMADs), including SMAD1, SMAD2, and SMAD3, thus activating the TGF-β target genes. Although the importance of USP15 in pathologic processes remains ambiguous so far, in this review, we endeavor to summarize the literature regarding the relationship of the deubiquitinating action of USP15 with the proteins involved in the regulation of Parkinson’s disease, virus infection, and cancer-related signaling networks.

## 1. Introduction

The balance of ubiquitin conjugation and de-conjugation is a well-tuned mechanism dictating the intracellular fate of the substrate proteins. The decoration of proteins with ubiquitin is achieved by an enzymatic cascade involving ubiquitin-activating enzyme (E1), ubiquitin-conjugating enzyme (E2), and ubiquitin ligase (E3). Ubiquitin moiety is attached to the target protein via a covalent isopeptide bond between the carboxyl group of C-terminal glycine (Gly) residue of ubiquitin and the ϵ-amino group of internal lysine (Lys) residue of the substrate protein [1,2]. Moreover, a poly-ubiquitin chain is assembled through the addition of an ubiquitin moiety to the one of the seven Lys residues or the N-terminal methionine residue of the preceding ubiquitin, giving rise to the diversity in the poly-ubiquitin arrangement [3,4]. In order to use the tagged form of ubiquitin for protein destruction, the poly-ubiquitin chain is linked together via Lys-48 residue persuading substrate proteins to undergo 26S proteasomal degradation [5]. Besides, the attachment of poly-ubiquitin chain linked together using Lys residues at different positions, most prominently via the Lys-63 residue, has been demonstrated to be a multifaceted protein modification for regulating various signaling pathways involved in transcription, membrane protein trafficking, nuclear transport, autophagy, as well as immune responses [6].

Ubiquitination is a reversible process and the removal of ubiquitin moieties from substrate—termed deubiquitination—which is conducted by deubiquitinating enzymes (DUBs). Deubiquitination is suggested to be a proofreading mechanism to prevent the inappropriate proteolysis of proteins [7,8]. Deubiquitination is important for keeping the ubiquitinated proteins moving through the 26S proteasome unimpeded, as the unanchored (free) ubiquitin chains could probably serve as competitive inhibitors with respect to ubiquitin-tagged proteins and thus needs to be disassembled by DUBs. Additionally, deubiquitination is critical for maintaining the homeostasis of ubiquitins, as the existence of abundant cellular nucleophiles—such as glutathione—allows them to adventitiously react with E1~ and E2~ubiquitin thioesters, with this reaction resulting in quickly exhausting the pool of free ubiquitins [9]. Deubiquitination is particularly important for processing of precursor ubiquitin into its mature form, because the mature ubiquitin is capable of conjugating with protein substrates via its exposed C-terminus. On the other hand, the precursor forms of ubiquitin are mostly unable to do so because their C-terminal glycine residue at position 76 are linked in an intermolecular bridge to the certain ribosomal proteins or head-to-tail-linked ubiquitin multimers [8,10].

Based on the features of catalytic domains and functional similarities, DUBs are classified into five subfamilies. Ubiquitin-specific proteases (USPs), ovarian tumor proteases (OTUs), ubiquitin C-terminal hydrolases (UCHs), and Machado-Joseph disease proteases (MJDs) are categorized into papain-like cysteine proteases, while JAB1/MPN/Mov34 proteases (JAMMs) belong to zinc-dependent metalloproteases [10]. In this review, we focus on the ubiquitin carboxyl-terminal hydrolase 15 (also known as ubiquitin-specific-processing protease 15, USP15), which features two consensus sequences harboring a classical catalytic triad (Cys-His-Asp) [11]. The amplification of *USP15* gene has been previously identified in glioblastoma, breast cancer and ovarian cancer. Furthermore, the possible connection between USP15 and cancer progression came from the discovery that USP15 exerts its deubiquitinating activity to stabilize several proto-oncogene proteins, such as E3 ubiquitin ligase MDM2 and type I TGF-β receptor. On the contrary, proteins possessing the tumor-suppressing function have also been identified as substrates for USP15 deubiquitination, including p53, an inhibitor of NF-κB (IκBα) and adenomatous polyposis coli (APC). Thus, the conflicting functions of these USP15 substrates in cancer biology raise a question concerning whether USP15 is more likely to act as an oncogene or as a tumor suppressor. Besides, there is a paralog of USP15, called USP4, which has been identified as an oncogene. The overexpression of USP4 has been immunohistochemically detected in small cell tumors and adenocarcinomas, supporting the oncogenic potential of USP15 [12]. Here, we review how the USP15 domains function in deubiquitination of protein substrates, and comprehensively describe the connection between USP15 and signaling pathways associated with cancer and other diseases.

## 2. The Structure and Function of the USP15 Domains

### 2.1. The Catalytic Domain

The USP15 and all other USP members contain a catalytic triad with the Cys, His, and Asp/Asn residues representing the best-characterized part of the Cys protease families. Structural analyses of catalytic triads have elucidated that catalytic Cys residue performs a nucleophilic attack on the isopeptide bond between the ε-amino group of Lys residue and the C-terminus of the distal ubiquitin. The reactivity of catalytic Cys residue depends on juxtaposition of His residue, which reduces the pKa of catalytic Cys residue, thereby Cys residue will be deprotonated [13]. One study demonstrated that the Asp/Asn residue is required for polarization of His residue to stabilize the catalytic activity of DUBs [14]. In USP15, delineation of amino acid sequence of the catalytic domain reveals the existence of six conserved boxes and the catalytic triad composed of Cys, His, and Asp residues, which are localized at Box 1, Box 5, and Box 6, respectively [11]. The subsequent study further revealed that the presence of two Cys-X-X-Cys motifs within Box 3 and Box 4 is critical for formation of a zinc-binding motif, which is important for conformational stability [15]. The catalytic domain of the USP family shares a high degree of homology in ubiquitin-bound structures, with a right hand-like configuration (palm, thumb, and fingers). The catalytic cleft, containing the critical residues for binding the C-terminal tail of ubiquitin, is located between the palm and thumb, whereas the two Cys-X-X-Cys motifs, coordinating one zinc ion in ways that help to stabilize its zinc ribbon structure, is located at the tip of the fingers [16]. It is known that the majority of USPs contain these Cys-X-X-Cys motifs except for USP5, USP7, USP10, USP13, USP14, USP25, USP28, USP34, and USP39 [17]. Results from one recent study further demonstrated that, in the absence of zinc ion or when zinc binding is abrogated by a mutation in the Cys residue, the USP15 fails to cleave the poly-ubiquitin chains from substrate proteins [18]. Moreover, some non-conserved residues found in the interface of USPs catalytic core in complex with the distal ubiquitin might play a part in conferring stereochemical diversity in the ubiquitin-binding site [19].

### 2.2. The DUSP Domain

**Figure 1:**
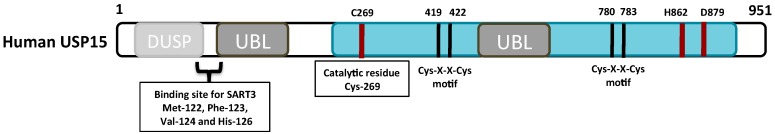
Schematic illustration of the domain organization of human USP15. Catalytic core (shown in cyan) is located in the carboxyl-terminal portion of USP15. The catalytic core of USP15 contains an inserted UBL domain. The USP15 with its active triad C269, H862, and D879 are denoted by thick red lines, and the Zn-coordinating Cys residues in positions 419, 422, 780, and 783 are highlighted by thick black lines. Four residues (Met-122, Phe-123, Val-124, and His-126) interacting with SART3 located between the amino-terminal DUSP domain and UBL domain are indicated. DUSP, domain present in ubiquitin-specific proteases; UBL, ubiquitin-like fold.

Apart from the catalytic domain, there is little or no conservation among the subdomains present in USPs [20]. The USP15 is comprised of multiple functional subdomains, including ubiquitin-specific protease (DUSP) domain and ubiquitin-like (UBL) domain, suggesting its ability in substrate recognition via protein/protein interaction [21]. As illustrated in Figure 1, human USP15 contains an N-terminal DUSP-UBL domain (DU domains) together with one UBL domain embedded in catalytic domain [20]. The multi-domain structure of USP15 with the position of catalytic triad and four Zn-coordinating Cys residues is indicated in Figure 1.

The USP15 is one of seven DUSP-containing USPs that is implicated in the regulation of COP9 signalosome [11,22]. The DUSP domain of USP15 is formed from approximately 120 amino acid residues, with a defined α1-β1-α2-β2-α3-β3 secondary structure. Using nuclear magnetic resonance (NMR) spectroscopy, the DUSP domain displays a “tripod”, a topologically complex structure that has a three α-helix bundle supporting a three-stranded anti-parallel β-sheet. There are four highly conserved residues between the helix α2 and strand β2, including Pro-62, Gly-63, Pro-64, Ile-65, called PGPI motif, which wrap around the aromatic side chain of another conserved Trp-37 residue located in the helix α2. This hydrophobic-packing interaction is important for stabilizing the DUSP structure [11]. Notably, analysis of surface hydrophobic residues of DUSP domain reveals two regions—named cluster A and cluster B—which may participate in substrate recognition by USP15. One region, containing residues Leu-32, Leu-79, Leu-83, Ile-84, Tyr-89, Ile-113, Arg-115, and Val-118, forms the highly conserved hydrophobic cleft located near the top face of its tripod-like structure. The second region, containing residues Leu-24, Trp-30, Phe-38, Phe-47, Ile-90, and Leu-91, is located between helix α1 and helix α2 and is also found to be conserved in the closest USP15 homologs, USP4 and USP11 [11].

### 2.3. The UBL Domain

Apart of catalytic domain, the ubiquitin-like (UBL) domain represents a common domain in the USP family. By comparing the secondary structure of all known USPs, it is observed that the one or multiple UBL domains are present in 18 human USPs, including USP4, USP6, USP7, USP9X/Y, USP11, USP14, USP15, USP19, USP24, USP31, USP32, USP34, USP40, USP43, USP47, USP48, and USP52 [14]. Although there is only little sequence homology among the UBL domains except for USP14 and USP48, they all share the ubiquitin-like topology and thus are sometimes called ubiquitin-fold domains (UFDs). Among these approximately 45–80 residues of UBL domains, the structural feature important for the activity and specificity of USPs is the β-grasp fold, which resembles ubiquitin but without the last two Gly residues that are required for ubiquitin conjugation [20].

**Figure 2:**
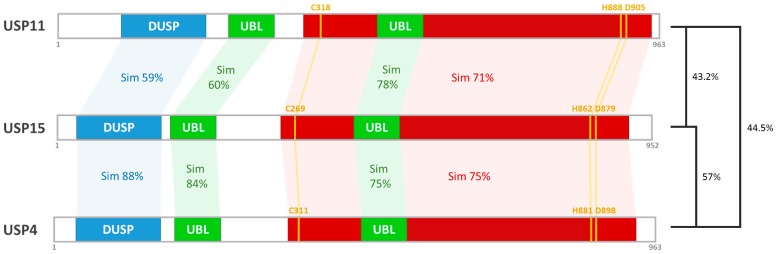
Alignments of USP15 and its paralogs USP4 and USP11. Paralogs of USP15, USP4 (NP_003354.2), and USP11 (NP_004642.2), show high similarity and identity in domain structure with USP15 proteins. Overall, USP15 shares 57% identity with USP4, and 43.2% identity with USP11. DUSP, domain present in ubiquitin-specific proteases; UBL, ubiquitin-like fold; red color represents USP catalytic domain; Sim, protein sequence similarity.

The presence of UBL domains both inside and outside of their catalytic core is likely to modulate the USPs function in enzymatic activity, recognition of different ubiquitin chain types, as well as recruitment of ubiquitinated proteins to the proteasome. For example, USP4, USP11, and USP15—the human USPs—possess an N-terminal UBL domain with another UBL domain lying within their catalytic domains. Besides, the UBL domains found in USP15 share approximately 60% or higher protein sequence similarity with those in USP4 and USP11 (Figure 2) [23]. More specifically, the insertion of the latter UBL domain is confined to the region in the middle of two Cys-X-X-Cys motifs that constitute the zinc-finger ribbon. Moreover, the location of the UBL domain—which is embedded within each USPs catalytic domain—was found to be homologous [21]. All such inserts arise through the inclusion of the almost intact UBL domain with variously sized residues (around 170–240 residues) following their C-terminus.

However, the regions surrounding the UBL-containing insert are responsible for forming a zinc-finger ribbon that is capable of binding to ubiquitin [24]. This phenomenon may play a role in the selective recognition of different types of ubiquitin chains. For instance, evidence for the selective recognition between monomeric ubiquitin and poly-ubiquitin chains has been found by using *o*-phenanthroline (OPT), a metal-chelating agent, which has the ability to remove the zinc ion binding in USP15. OPT inhibits the degradation of K48-linked poly-ubiquitin chains but does not affect the deubiquitinase activity of USP15 toward ubiquitin-GFP fusion protein. Such USP15 loses its subtle ability to recognize the certain ubiquitin linkage types, partly because its zinc-finger domain, which includes a large (~330 residues) UBL-containing insert, becomes dysfunctional [15,18].

Because very little is known about the functions of UBL domains in USP15, some of the cases described below provide only circumstantial evidence supported by other annotated USPs. A good example is USP4, the closest paralog of USP15 that shares 76% sequence similarity [25], containing two UBL domains in the same region of the USP15. The UBL domain embedded in the USP4 catalytic core acts as a competitive inhibitor of ubiquitin binding to USP4 catalytic core in an auto-regulatory manner [20,26]. By comparing the crystal structure of truncated USP4 (an intact catalytic domain but without the upstream N-terminal region) to the other USPs, it is observed that the embedded UBL domain self-associates well with its catalytic core [26].

Unlike the N-terminal UBL domains found in USP9X/Y, 14, 34, and 47, the N-terminal UBL domains found in the three closely related USP4, 11, and 15 are preceded by the DUSP domain. Classical UBL domains display a ubiquitin-like β-grasp fold, whereas, in the presence of DUSP domain, the UBL domain interacts more favorably with adjacent DUSP domain and thereby forms a β-hairpin structure (DU finger) at their interface via hydrogen-bonding interactions [27,28]. This DU finger present in both USP4 and USP15 is responsible for the binding of squamous cell carcinoma antigen recognized by T-cells 3 (SART3), which is considered to be a U4/U6 small nuclear RNA recycling factor and also a tumor antigen usually recognized by the HLA-A24-restricted and tumor-specific cytotoxic T lymphocytes. The SART3 recognition by USP4 and USP15 is mainly relying on four amino acid residues, i.e., L126, F127, V128 and H130 and M122, F123, V124, and H126. However, the USP11 also possesses the N-terminal DU finger but lacks the three critical residues G187, L/M188, and F189, leading to disability to bind to SART3. This suggests that the presence of N-terminal DUSP and UBL domains might play a delicate role in specific recognition of their protein substrates. Interestingly, deletion of the nuclear localization signal (NLS) sequence in SART3 concomitantly abolishes the translocation of both USP4 and USP15 from the cytosol to the nucleus, particularly where USP4 and USP15 are capable of exerting their deubiquitination activities [29,30].

## 3. Chromosomal Location and Isoforms of USP15

The *USP15* gene is located on the chromosome band 12q14.1 and spans 149,382 bases of genomic DNA. Besides, there is a pseudogene of USP15 located on the long arm of chromosome 2. *USP15* gene encodes 19 transcripts (splice variants) but only eight of them contain the coding region. There are only three transcript models having a high level of support through the full length of their exon structure described by Ensembl genome database (Table 1). The protein length of human USP15 contains 981, 952, and 235 amino acid residues in isoform-1, -2, and -3, respectively. On the other hand, the mouse ortholog of USP15 is shown to have 98% amino acid similarity with human USP15. Mouse USP15 shares 75.5% sequence similarity with the mouse USP4, named Unp, which has been identified as a proto-oncogene able to promote tumor initiation when overexpressed in nude mice [31].

## 4. Expression and Subcellular Localization of USP15 Protein

The mRNA of human USP15 is found ubiquitously in various organs and tissues. The protein levels of USP15 are most abundant in the testes, pancreas, thyroid gland, and adrenal gland (Table 2) in line with the observation shown in mouse Usp15 and the rat ortholog UBP109. USP15 harbors both nuclear export signals (NESs) and nuclear localization signals (NLSs), however, its cellular distribution is not absolutely determined by NES and NLS signals alone [32]. For example, in NIH3T3, mouse fibroblast USP15 resides in the cytosol while in MDA-MB-231 human breast cancer cell line, USP15 resides close to the plasma membrane. Interestingly, the cellular localization of USP15 and USP4 in HeLa human cervical cancer cells is mutually exclusive of one another. USP15 is mainly detected both in the cytosol and in the nucleolus, while USP4 is only observed in the nucleus. These results suggest that although USP4 and USP15 share high structural and functional homology, their biological functions take place in different cellular compartments [33]. Moreover, one report demonstrated that the cellular localization of USP15 is determined by its interacting partner, SART3 [34].

## 5. USP15 and Oncogenic Signaling

USP15 has been implicated in a variety of cellular signaling events, including the COP9-signalosome [18], TGF-β [35,36], NF-κB [37], β-catenin [22], and p53 signaling pathways [38]. All of these pathways have been reported to have a potent oncogenic activity. For example, USP15 protects the component subunit of cullin-RING ubiquitin ligase (CRL) from auto-ubiquitination and degradation [18,22]. USP15 is known to promote stabilization of the TGF-β receptor and its downstream signal transducers, known as receptor-activated SMADS (R-SMADS), thus empowering the TGF-β signaling [35,39]. Another report reveals that USP15 deubiquitinates IκBα and increases its re-accumulation, leading to reduced NF-κB activity [37]. Other proteins involved in oncogenic pathways mediated by USP15 have also been described. The mechanisms underlying the deubiquitination of these proteins and the status of their respective pathways are discussed in the following sections.

### 5.1. COP9-Signalosome/Cullin-RING E3 Ubiquitin Ligase

The COP9-signalosome (CSN), a conserved eight-subunit (CSN1–8) protein complex, has been found to be involved in the ubiquitin-proteasome pathway in all eukaryotic cells. CSN serves as a dominant regulator of cullin-RING ligase family of ubiquitin E3 complexes (CRLs) [40]. However, it was reported that Ubp12p, a *Schizosaccharomyces pombe* ortholog of human USP15, was able to interact with CSN as observed by systematic mass spectrometry screening [41]. Previous studies indicated that USP15 cooperates with CSN to regulate the ubiquitination status and stability of CRLs [18]. CRLs form a group of highly polymorphic E3 ligases composed of four core subunits as follows: (1) a central scaffold consisting of one of seven cullin isoforms; (2) a catalytic component called RING BOX protein -1 or -2 (RBX1 or RBX2) that contains a zinc-binding consensus sequence cooperating with CULs scaffold to maintain the ligase activity of CRL; (3) a substrate recognition component built up from one of the large F-box protein families that provides a great diversity of cullin-based E3 ligases to target protein substrates; (4) an adapter component termed Skp1, which is responsible for bridging the F-box protein to CRL complex [42].

The biochemical activity of CSN is specified by its isopeptidase activity, which catalyzes the removal of the ubiquitin-like protein Nedd8, termed deneddylation, from cullin subunits of CRL complexes and transiently suppresses the E3 ubiquitin ligase activity [42]. Moreover, the CSN-mediated deneddylation also impedes the protein turnover rates of CRLs. This phenomenon is explained by two different ways. One way is mediated by neddylation status of CRL, where the lack of Nedd8 tags on CRL results in a concomitant loss of auto-ubiquitination activity towards CRL itself, thereby preventing CRL from proteasomal degradation [40,43]. The other mechanism was described as cooperation between USP15 and CSN, which reverses auto-ubiquitination of CRL component, RBX1, thereby stabilizing the assembled CRL complex while retaining its E3 ubiquitin ligase activity [18,44]. Besides, the CSN plays a significant role in many cancer-associated pathways including the cell cycle, repair and DNA damage sensitivity, and apoptosis [45,46]. The functions of CSN, CRLs, and their corresponding E3 substrates are frequently dysregulated in a number of cancers, reflecting a participation of these proteins in tumor progression [47].

### 5.2. COP9-Signalosome/The IκBα-Mediated Regulation of NF-κB

A study has shown that USP15 cooperates with CSN to stabilize the IκBα, leading to the inhibition of NF-κB signaling. In this case, the detailed mechanism of IκBα stabilization involves the inhibition of the E3 ubiquitin ligase activity of CRL/SCF^β−TrCP^, which has a potential role in recognizing the phosphorylated IκBα at Ser-32 and Ser-36 residues for ubiquitin-dependent degradation. However, more evidence for such stabilization should be obtained [37,48].

### 5.3. COP9-Signalosome/The APC-Mediated Regulation of β-Catenin Stability

Loss-of-function mutation in adenomatous polyposis coli (APC) tumor suppressor or gain-of-function mutation in β-catenin oncogene have long been recognized to lead to constitutively active Wingless/Int-1 (Wnt) signaling, giving rise to a high risk of colorectal cancer development [49]. Disrupting the balance between β-catenin and APC frequently also results in cancer progression by driving cell transformation, tumor angiogenesis, and metastasis [50]. The current study suggested that the cooperation between CSN and USP15 is implicated in negative regulation of Wnt signaling by supporting the build-up of β-catenin destruction complex, which is composed of axin, casein kinase 1α, and glycogen synthase kinase 3β and requires APC to recruit phosphorylated β-catenin. In the absence of Wnt signals, β-catenin is sequestered by such a complex and becomes ubiquitinated by CRL/SCF^β−TrCP^ ubiquitin ligase. However, it has been reported that the assembly of β-catenin destruction complex is attributed to CSN-mediated deneddylation and also reliant on CSN-associated USP15, which catalyzes deubiquitination of APC [22]. Besides, the APC is also involved in mitotic dimension through interaction with the microtubule end-binding protein (EB1) that controls microtubule dynamics and microtubule-dependent processes [51]. Unlike the mechanism of USP15-mediated stabilization of APC, USP15 destabilizes EB1. This is due to the fact that EB1 is not a direct substrate of USP15. USP15 acts via stabilization of CRL, which accelerates ubiquitination and leads to degradation of EB1 [52].

### 5.4. Transforming Growth Factor β (TGF-β) Signaling Pathway

**Figure 3:**
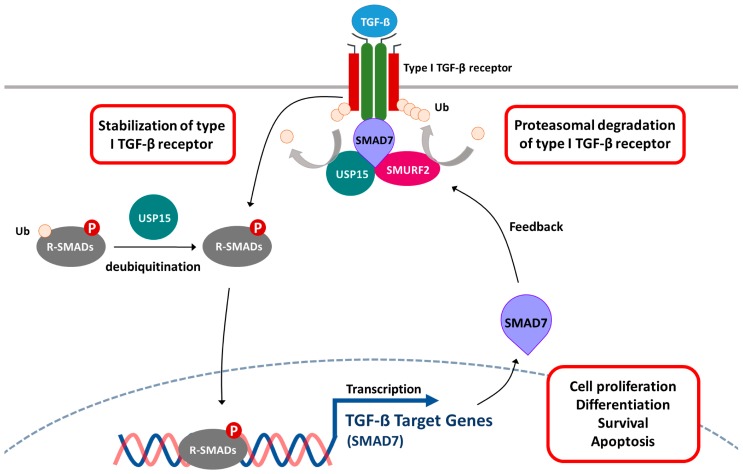
USP15 acts as a “biological thermostat” in the TGF-β signaling pathway. USP15 competes with the action of SMAD7-SMURF2 complex that trends to promote the ubiquitination of type-1 TGF-β receptor. USP15 participates in deubiquitination of the monomeric ubiquitin from R-SMADs, which participates in transcription of TGF-β target genes. Protein ubiquitination is indicated by the presence of an attached ubiquitin moiety (Ub). The phosphate group (P) is shown as a red sphere.

The TGF-β is a secreted cytokine implicated in embryonic development, tissue homeostasis, wound healing, cancer progression, and immunity. The oncogenic role of TGF-β has also been reported and suspected to be a therapeutic target in advanced cancer [53]. Recent reports have designated USP15 as a “biological thermostat” of the TGF-β pathway, because it is capable of subtle modulation of the TGF-β activity (Figure 3) [35,54]. Briefly, TGF-β signaling pathway is initiated by the formation of a ligand-induced heteromeric complex in which the type I (signal transducer) receptor is activated by the type II (activator) receptor via serine/threonine phosphorylation events. The activated type I receptor transmits the intracellular signals through phosphorylation of receptor-regulated SMADs (R-SMADs), particularly SMAD2 and 3, on their C-terminus. Despite the TGF-β activity being tightly regulated by phosphorylation, its activity is also ubiquitin-dependent [55,56]. As for the negative feedback control, SMAD7 acts as a scaffold protein to recruit SMURF2, an E3 ubiquitin ligase responsible for ubiquitination of TGF-β receptor complex [57,58]. On the contrary, USP15 plays an antagonistic role in SMAD7-SMURF2 complex-mediated ubiquitination of type I TGF-β receptor (TβR-I) by detaching the ubiquitin tags from TβR-I [35]. It has also been reported that USP15 is involved in the regulation of R-SMADs activity that is dependent on the presence or absence of mono-ubiquitin decoration. USP15 removes the mono-ubiquitin tags from R-SMADs and rescues its ability to recognize its target promoter on DNA [39]. The overexpression of the *USP15* gene results in the dysfunction of the TGF-β pathway, and has been found in several types of cancer, such as glioblastoma, breast, and ovarian cancer [35].

### 5.5. The Regulation of Mouse Double Minute 2 Homolog (MDM2) and p53

Mouse double minute 2 homolog (MDM2), a well-characterized proto-oncogene, is an E3 ubiquitin-protein ligase that mediates the tumor suppressor p53 leading to proteasome degradation [59,60]. Overexpression of MDM2 protein, owing to gene amplification as well as transcriptional and posttranslational regulation, has been associated with more than 40 different types of malignancies [61]. In the recent study, predominant expression of USP15 was found to be associated with a decrease in tumor cell apoptosis and antitumor T cell response, as evidenced by preventing ubiquitin-dependent degradation of MDM2 in melanoma and colorectal cancer cell lines [38]. Nevertheless, considering that USP15 maintains MDM2 protein level and negatively regulates p53-dependent gene transcription and apoptosis in cancer cells, USP15 may represent an excellent drug target for efficient cancer therapy [18,38].

### 5.6. Regulation of p53 through Stabilizing HPV/E6 Oncoprotein

Human papillomaviruses (HPVs), a large heterogeneous virus family containing more than 100 types, harbor E6 zinc-finger oncoprotein that gives rise not only predominately to human cervical carcinoma, but is also implicated in the development of anogenital, head and neck, and cutaneous cancers [62]. The presence of high-risk HPVs, E6 protein is frequently found in HPV-positive cancers. E6 protein abrogates the tumor suppressive role of p53 and the impact of E6 protein is unequivocally related to cell cycle alterations, protection from apoptosis, and transformation [63]. USP15 has been reported to interact with HPV type-16 E6 oncoprotein and regulate its stability. Knockdown of USP15 by siRNA approach demonstrated that silencing of USP15 decreases the protein levels of E6 significantly. On the contrary, overexpression of USP15 increases the steady-state levels of E6, suggesting E6 protein could be a target for USP15-directed deubiquitylation [64]. Interestingly, a study reported that the siRNA-mediated USP15 knockdown led to reduction in E6 level while the expression of p53 was not increased, suggesting the involvement of an additional compensatory mechanism in the modulation of p53 levels [65].

## 6. Acquired Chemoresistance of Cancer Cells

Paclitaxel (also known as Taxol), an antitumor agent originally isolated from the Pacific yew tree (*Taxus brevifolia*), shows encouraging clinical activity in patients with solid tumors including breast, ovarian, prostate, and non-small-cell lung cancers. It is one of the most powerful chemotherapeutic agents that inhibit the capability of depolymerizing microtubules, thereby retarding and even halting cancer cell division [66]. Despite the favorable prognosis observed after the initial treatment by paclitaxel, some patients still had a high risk of unexpected development of progressive disorders. The occurrence of paclitaxel resistance is the major obstacle to the improvement of therapeutic response and survival in paclitaxel-treated cancer patients [67]. Thus, an in-depth investigation of the molecular mechanisms dedicated to drug resistance can offer the opportunity to expand more effective anticancer therapies.

A variety of cell death phenomena have been associated with paclitaxel, ranging from interrupting microtubule dynamics to cell cycle arrest at the G2/M phase and promoting the serial cleavage events of apoptotic effector caspases (CASPs), such as caspase-10, -8, -7, and -3 [68,69,70]. A report suggested that paclitaxel sensitivity has a positive correlation with USP15 protein expression in HeLa cells, and that the binding of procaspase-3 with the components of SCF (Skp1-CUL1-F-box) may be controlled by USP15. It may be partly due to ubiquitination of procaspase-3 by SCF E3 ubiquitin ligase, which reduces the basal level of procaspase-3 and protects cells from apoptosis through increased apoptosis threshold [71]. Therefore, the authors proposed that, under the sufficient levels of USP15 protein present in paclitaxel-treated cells, procaspase-3 tends to be deubiquitinated and stabilized, thereby offering higher susceptibility to paclitaxel-induced apoptosis. This suggests that the expected outcome of paclitaxel-treated subjects may be predicted from USP15 measurements [72].

## 7. Diseases Associated with Dysregulation of USP15

### 7.1. USP15 and Parkinson’s Disease

The most frequent cause of recessive Parkinson’s disease (PD) is loss-of-function mutations in *PARK2* gene, which encodes the E3 ubiquitin ligase, Parkin. Parkin is present in the cytosol under normal circumstances, and is dictated to translocate to the depolarized mitochondria for ubiquitination of the outer mitochondrial membrane proteins and for induction of mitophagy, a process characterized by the selective removal of damaged mitochondria through autophagy [73].

In the PD cases with extremely reduced Parkin activity, inhibition of USP15 appears to rescue the defect in Parkin-mediated mitophagy. However, the actions of USP15 on blocking mitophagy seem to be mainly caused by counteracting Parkin-mediated mitochondrial ubiquitination [74]. This observation also indicated that Parkin-mediated mitophagy was only antagonized by USP15 but not the other closely related USP4 and USP11. Besides, loss-of-function mutations of PARK2 and decreased expressions of Parkin have been reported in glioblastoma and other human cancers [75]. Thus, the opponent relationship of Parkin and USP15 shed light not only on physiological aspects of PD pathogenesis but also on tumorigenesis [74].

### 7.2. USP15 as Regulator of Antiviral Innate Immune Responses

Upon virus infection, infected cells possessing a viral sensor, a RNA helicase called retinoic acid-inducible gene-I (RIG-I), which elicits the innate immune response through recognizing the accessible phosphate groups present at the 5′-termini of viral RNA. The types of virus that could be discriminated by RIG-1 include the influenza virus, paramyxoviruses, flavivirus, and the rhabdovirus vesicular stomatitis virus (VSV) [76]. Notably, activation of RIG-1 is thought to be dependent on ubiquitin modification, represented by Lys-63-linked polyubiquitin chains, leading to the production of antiviral cytokines, interferon-α and -β (IFN-α/β) [77]. This type of RIG-1 ubiquitination is apparently attributable to ubiquitin E3 ligase tripartite motif protein 25 (TRIM25). However, USP15 has been demonstrated to play a regulatory role in TRIM25-RIG-1 antiviral signaling. By deubiquitinating TRIM25, USP15 ensures that TRIM25 will not be tagged with Lys-48-linked ubiquitin chains and thus will not be degraded by proteasome [78]. The presence of USP15 sustains the antiviral immune response by stabilizing TRIM25 proteins following recognition of viral RNA. In addition, targeting USP15 may provide a complementary approach for the treatment of autoimmune disease by preventing the redundant production of IFNs and pro-inflammatory cytokines [79].

## 8. Concluding Remarks

Deubiquitination serves as a proofreading mechanism to improve the accuracy of tagging proteins for destruction. Moreover, such removal of ubiquitin tags is also the means of regulating the biological activities of the protein substrates. Various DUBs have been identified as crucial regulators of transcription, membrane protein trafficking, nuclear transport, autophagy, as well as immune responses [4]. Notably, inappropriate ubiquitin modification is often associated with diseases, such as Parkinson’s disease, virus infections, and certain types of cancer, thus the particular ubiquitin-modified protein substrates could be used to study the biological significance of ubiquitin ligases or DUBs. There is a lack of research on the mechanisms of DUBs as compared to the extensive literature available regarding the ubiquitin ligases. However, results from some of the studies proved that USP15 possesses oncogenic potential for the promotion of glioblastoma and breast cancer via activation of the TGF-β pathway [35,39]. Moreover, the literature describes the role of USP15 in the deubiquitination and stabilization of oncoproteins, MDM2 and HPV type-16 E6, thus abolishing the tumor suppressor activity of p53 [38,64]. In contrast to the evidence supporting the oncogenic role of USP15, several studies uncovered that USP15 cooperates with COP9-signalosome (CSN) to stabilize the critical negative regulators of oncogenic NF-κB and Wnt pathways, IκBα and APC, respectively [22,37]. Therefore, in order to better understand the role of USP15, extensive research is required. Comprehensive approaches such as proteomics are urgently needed to identify the entire set of protein substrates that are recognized by USP15, which would help us to understand the mechanisms by which USP15-mediated deubiquitination affects cancer and other diseases.

## Figures and Tables

**Table 1:** Summary of three human USP15 mRNA transcripts and protein isoforms described by Ensembl genome database (available on: http://asia.ensembl.org/index.html) and UniProt database (available on: http://www.uniprot.org/uniprot/Q9Y4E8).

mRNA Transcripts	Transcript ID	Length (bp)	Exon	Protein Isoform	Protein ID	Protein Size (aa)	Mass (kDa)
USP15-001	ENST00000353364	14,950	21	ubiquitin carboxyl-terminal hydrolase 15 isoform 2	NP_006304	952	109.297
USP15-002	ENST00000280377	4,748	22	ubiquitin carboxyl-terminal hydrolase 15 isoform 1	NP_001239007	981	112.419
USP15-003	ENST00000312635	2,226	7	ubiquitin carboxyl-terminal hydrolase 15 isoform 3	NP_001239008	235	27.094

**Table 2:** Tissue expression of human USP15 provided by Human Protein Atlas database (available on: http://www.proteinatlas.org). The signal intensity of USP15 protein levels was scored as follows: **-**, negative; **+**, low; **++**, moderate; **+++**, high.

	Organs	Protein Expression Overview (Score)		Organs	Protein Expression Overview (Score)
Adipose tissue/Soft tissue	Adipose tissue	**+**	Immune system	Appendix	**+++**
	Soft tissue	**++**		Bone marrow	**++**
Brain	Cerebral cortex	**+**		Lymph node	**++**
	Hippocampus	**+**		Tonsil	**++**
	Caudate	**+**		Spleen	**++**
	Cerebellum	**+**	Kidney	Kidney	**++**
Endocrine tissues	Thyroid gland	**++**		Urinary bladder	**+**
	Parathyroid gland	**+++**	Liver/Gallbladder	Liver	**+**
	Adrenal gland	**++**		Gallbladder	**+++**
Female tissues	Breast	**+**			
	Vagina	**-**	Lung	Nasopharynx	**++**
	Cervix, uterine	**+**		Bronchus	**+**
	Endometrium	**+**		Lung	**++**
	Fallopian tube	**++**	Male tissues	Testis	**++**
	Ovary	**+**		Epididymis	**++**
	Placenta	**++**		Prostate	**++**
Gastrointestinal tract	Oral mucosa	**+**		Seminal vesicle	**++**
	Salivary gland	**+**	Muscle tissues	Heart muscle	**+**
	Esophagus	**+**		Skeletal muscle	**++**
	Stomach	**++**		Smooth muscle	**++**
	Duodenum	**+++**	Pancreatic tissues	Pancreas	**++**
	Small intestine	**+++**	Skin	skin	**++**
	Colon	**++**			
	Rectum	**+++**			
