# Correction: Chon-Kit Chou, et al. The Regulations of Deubiquitinase USP15 and Its Pathophysiological Mechanisms in Diseases. *Int. J. Mol. Sci.* 2017, *18*, 483

**DOI:** 10.3390/ijms18050902

**Published:** 2017-04-25

**Authors:** Chon-Kit Chou, Yu-Ting Chang, Michal Korinek, Yei-Tsung Chen, Ya-Ting Yang, Steve Leu, I-Ling Lin, Chin-Ju Tang, Chien-Chih Chiu

**Affiliations:** 1 Graduate Institute of Natural Products, Kaohsiung Medical University, Kaohsiung 807, Taiwan; fatchou1988@hotmail.com (C.-K.C.); mickorinek@hotmail.com (M.K.); 2 Department of Biotechnology, Kaohsiung Medical University, Kaohsiung 807, Taiwan; c.jasmine8306@gmail.com (Y.-T.C.); tinayang101@gmail.com (Y.-T.Y.); st.leu.tw@gmail.com (S.L.); candace0602@gmail.com (C.-J.T.); 3 Department of Medicine, Yong Loo Lin School of Medicine, National University of Singapore, Singapore 117, Singapore; mdccyt@nus.edu.sg; 4 Institute for Translational Research in Biomedicine, Kaohsiung Chang Gung Memorial Hospital, Kaohsiung 807, Taiwan; 5 Department of Medical Laboratory Science and Biotechnology, Kaohsiung Medical University, Kaohsiung 807, Taiwan; linili@cc.kmu.edu.tw; 6 Center of Excellence for Environmental Medicine, Kaohsiung Medical University, Kaohsiung 807, Taiwan; 7 Graduate Institute of Medicine, College of Medicine, Kaohsiung Medical University, Kaohsiung 807, Taiwan; 8 Translational Research Center, Cancer Center and Department of Medical Research, Kaohsiung Medical University Hospital, Kaohsiung 807, Taiwan; 9 Department of Biological Sciences, National Sun Yat-sen University, Kaohsiung 804, Taiwan

We would like to make a clarification to our article [1] entitled “The Regulations of Deubiquitinase USP15 and Its Pathophysiological Mechanisms in Diseases”. We regret that cited reference 26 [2] in the paper has been retracted [3]. Luna-Vargas et al., retracted the paper because they could not reproduce the results of lower *K*_m_ for the USP4 catalytic domain under their described conditions [2]. Despite their article withdrawal, they emphasized that the USP4 crystal structure and the ubiquitin-binding data in [2] are not in question. To avoid any confusion of the readers, we have replaced the above reference with a new reference [4] and rephrased the statement in the article. We would like to apologize for any inconvenience caused.

The manuscript will be updated, and the original will remain online at the article webpage.
